# Exogenous diatoms ameliorate thermal bleaching of symbiont bearing benthic foraminifera

**DOI:** 10.1098/rspb.2025.0596

**Published:** 2025-06-18

**Authors:** Danna Titelboim, Craig J. Dedman, Rose Pian Hodgson, Lucy S. Knowles, Xuan Liu, Luca Lenzi, Jack Tudor, Edith Vamos, Rosalind E. M. Rickaby

**Affiliations:** ^1^ University of Oxford, Oxford, UK; ^2^ University of Sheffield, Sheffield, UK; ^3^ University of Liverpool, Liverpool, UK

**Keywords:** warming, thermal tolerance, bleaching, large benthic foraminifera, diatom symbionts

## Abstract

Many marine calcifiers engage in obligatory algal symbiosis which is threatened by ocean warming. Large benthic foraminifera are prominent carbonate and sand producers in shallow environments with a wide range of species-specific thermal tolerances assumed to be related to their diverse algal symbionts. We examine two diatom-bearing benthic foraminifera species which differ in their thermal physiological tolerance and symbiont community composition. Our findings demonstrate that the less thermally tolerant host, *Amphistegina lobifera* Larsen, 1976, ‘shuffles’ the dominant players of the internal symbiont community with increasing temperature while the more thermally tolerant host *Pararotalia calcariformata* McCulloch, 1977, is dominated by *Arcocellulus cornucervis* Medlin, 1990, at all temperatures. Although this diatom species was present in *A. lobifera* from all treatments, it became more abundant only under the most severe temperature stress. Symbionts were isolated from the thermally tolerant foraminifera *P. calcariformata*, with only one species of symbiont surviving at 35°C, while the others failed to survive at 32°C. Supplementation of isolated symbionts reduced bleaching of *A. lobifera* under heat stress suggesting that while increased temperature creates shuffling at the family level, heat tolerance of the holobiont is related to changes at the species level of the symbiont algae.

## 1. Introduction

Climate change poses a threat to marine calcifiers, crucial ecosystem engineers and a key part of the marine carbon cycle. Many of these organisms engage in obligatory algal symbiosis that supply the host with products of photosynthesis, providing energy that supports calcification [1]. These symbioses are especially beneficial in oligotrophic environments where nutrient cycling between host and symbionts becomes crucial for their survival [2,3]. Changes in symbiont community composition and abundances can support the tolerance of a host to environmental stressors as more diverse symbiont communities can provide an ecological advantage under different or changing conditions [4]. Thus, the flexibility of a host to accommodate diverse symbionts and manipulate the internal community, or to acquire new symbionts from the ambient water could play a key role in their ability to survive environmentally induced stress [5,6].

Corals host diverse genera of Symbiodiniaceae but are often dominated by just a single species that is responsible for the thermal sensitivity of the holobiont [7]. There are many examples of changes in the relative abundance of Symbiodiniaceae communities within the host (known as ‘shuffling’) in response to environmental change. Reports of uptake of new symbiont partners from the ambient water (known as ‘switching’) in adult corals remain limited and in most cases it is unclear if in fact the ‘switched’ strains of symbionts may have been present in low abundance beneath the detection limit of metabarcoding methods [8,9]. Only recently, Chan *et al*. [10] were the first to demonstrate that heat-evolved symbionts can be acquired by adult corals, and are able to persist long term within the host, such that coral thermotolerance is enhanced without any trade-off with growth. This holds great promise in the path to mitigate the effect of future warming on coral reefs. But this has only been observed in one coral species (*Galaxea fascicularis*) so any implementation of introducing heat-evolved symbionts as a strategy in large-scale coral restoration projects will require a greater understanding of what allows the uptake of different symbionts and the mechanism of enhanced thermal tolerance. Environmental uptake of exogenous symbiont algae has also been demonstrated in sea anemones [11] and octocorals [12], but not in any calcifying organisms that could provide insight on the underlying mechanisms through which algal symbionts support the heat tolerance of their calcifying hosts, which play prominent roles in producing carbonate structurers and sands.

Large benthic foraminifera (LBF) are prominent carbonate producers in shallow environments [13,14] and a main source of sands, stabilizing coastlines in many islands [15]. LBF have a wide range of species-specific thermal tolerances, with some species exhibiting extreme thermal resilience, calcifying even at 40°C [16,17]. They were also successful through past warming events, even when other calcifiers disappeared (e.g. the Palaeogene ‘reef gap’ [18–20]). This high tolerance in both modern and past species, especially when compared with today’s most dominant shallow water calcifiers, implies that their role as ecosystem engineers will become more prominent as warming progresses, making it imperative to understand how they will be affected. Further, LBF have a wide range of species-specific thermal tolerances that are suggested to be related to their flexibility in algal symbiosis [21,22] that can involve diatoms, dinoflagellates, chlorophytes or unicellular rhodophytes [23]. In general, the variations in evolutionary lineages of the endosymbionts correspond to the phylogenetic relationships between the host organisms. The key factor controlling the composition of the symbiont community is their local habitat [24,25]. This provides an opportunity to explore questions of host specificity and host–symbiont interactions which could be the key to understanding LBF resilience to environmental stressors, and the mechanisms in which photosynthesis supports calcification.

In this study, we examine two foraminifera species demonstrating different species-specific thermal tolerances: *Amphistegina lobifera* and *Pararotalia calcariformata. Amphistegina lobifera* is a very common species abundant across a wide geographical range even though it is limited by a well-defined temperature range [26]. Their thermal optimum for calcification is 20–25°C with complete inhibition at both 15°C and 35°C, while net photosynthesis remains constant across 15–32°C [27,28].

*Pararotalia calcariformata*, on the other hand, continues to calcify up to at least 36°C [16] with high photosynthetic efficiency up to 35°C [29]. In terms of their symbionts, both host species are diatom bearing but dominated by different diatom families [21,28]. In this study, we examine how the internal symbiont community of both species changes in response to thermal stress, and whether introduction of a more thermally tolerant symbiont can confer a greater thermal tolerance to the host.

## 2. Methods

**Figure 1.**
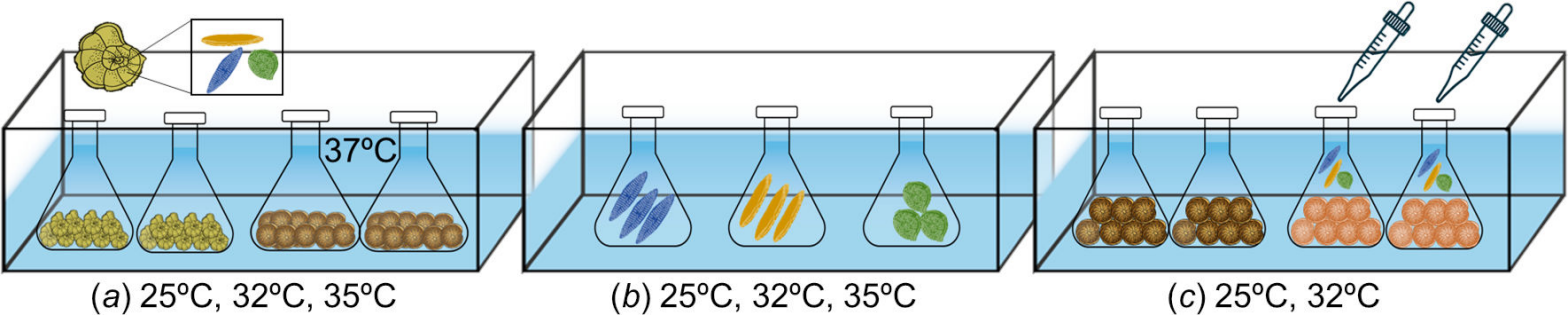
Experimental design: (*a*) Symbiont shuffling experiment examining changes in the internal symbiont community with increasing temperatures up to 35°C for *P. calcariformata* and 37°C for *A. lobifera*. (*b*) Isolated symbiont experiment assessing the thermal tolerance of isolated symbionts from *P. calcariformata*. (*c*) Symbiont switching experiment testing if thermal tolerance of *A. lobifera* can be enhanced by uptake of symbionts isolated from *P. calcariformata*. The colours of the holobionts in the figure demonstrate different symbiont species composition.

To examine temperature-related changes in symbiont community within a host and their effect on the holobiont thermal resilience we conducted three experiments: First, we assessed how the internal symbiont community in both species change with increasing temperatures induced in the laboratory (Symbiont shuffling experiment; figure 1*a*). We then isolate symbionts from the more tolerant host *P. calcariformata* (presumed to be similar to the natural symbiont community at the beginning of the shuffling experiment) and examined their thermal tolerance (Isolated symbiont experiment; figure 1*b*). Finally, we tested whether the lower heat-tolerant host (*A. lobifera*) after partial bleaching at 32°C, can recover following an addition of isolated symbionts at optimal and stressful temperatures (Symbiont switching experiment; figure 1*c*).

### (a) Symbiont shuffling experiments

Samples of foraminifera were collected by scraping algal mats from the coastal rock surface on the northern Mediterranean coast of Israel during June 2022. The samples were sieved between 150 and 1000 µm and transported to the laboratory for further analysis. Live specimens of *A. lobifera* and *P. calcariformata* were picked under a binocular microscope, cleaned and transferred to culturing bottles with calcein-spiked seawater (approx. 40 μM) and kept in 25°C for 10 days. Calcein was used to ensure specimens were healthy and actively calcifying before the experiment. Specimens that created new chambers (indicated by chambers with fluorescent calcite) were randomly divided into groups of 10 specimens from each species, and placed in 50 ml culturing flasks with artificial tropical seawater, partitioned into temperature-controlled water baths of 25°C, then increased by 1°C per hour to 32°C and ultimately 35°C. Specimens of *A. lobifera* were also exposed to an additional treatment of 37°C (slowly amplified from 25°C) to test the response of their symbionts to extreme stress. During the experiment, samples were kept under a 12:12 h light–dark cycle (approx. 45 μmol photons m^−2^ s^−2^). Water was replaced weekly and each time salinity (41) and pH (8.1) were monitored, and foraminifera were fed with 30 µl autoclaved marine microalgae Nannochloropsis food mix (following [30]). After 30 days, specimens were briefly examined for pseudopodal activity and 10 specimens (randomly chosen from two culturing flasks) from each treatment were immediately frozen at −80°C and stored for further analysis of their algal symbiont community.

### (b) Isolation of symbionts and thermal resilience characterization

Three symbiont strains were isolated from an individual specimen of *P. calcariformata* collected by scraping algal mats from the coastal rock surface on the northern Mediterranean coast of Israel. Symbionts were isolated following a protocol adapted from [31]. Briefly, foraminifera were brushed and cleaned in sterile synthetic ocean water (SOW) and crushed using sterilized fine-pointed tweezers in 0.2 µm filter-sterilized SOW with F/2 + Si nutrients [32]. The isolated culture was placed in a PHCbi MLR-352 Climate Chamber maintained at 23°C (14 : 10 light : dark cycle; 60−80 μmol m^−2^ s^−1^) until algal growth was visible. Single algal colonies were isolated by use of 2% agar plates (F/2 + Si) and monocultures established in 20 ml fresh medium. Antibiotic treatment (as described by Amin *et al*. [33]) was used to remove bacteria. Isolated strains were routinely transferred to fresh growth media upon reaching the mid-exponential phase of growth. Taxonomical identification of the symbionts was done according to taxonomical features of their frustules which they silicify after isolation from the host, visualized by SEM, and following [21,34].

To examine the thermal tolerance of the isolated symbionts, three replicates of each strain were inoculated in 20 ml artificial tropical seawater and placed in temperature-controlled water baths of 25°C, 32°C and 35°C, under a 12:12 light–dark cycle (approx. 45 μmol photons m^−2^ s^−2^). The cultures were then monitored daily for their cell count using a BD Accuri C6 Plus Flow cytometer, and once exponential growth rate was observed cultures were re-inoculated again to 20 ml. To account for acclimation time, specific growth rates were calculated from the logged growth curves of the third inoculation. Cultures were considered to not survive a treatment if cytometer counts were zero and no cells were observed by microscopy.

### (c) Symbiont supplementation experiment

Additional specimens from the shuffling experiment exposed to the 32°C treatment were maintained at the same temperature for another 45 days and when partly bleached (parts of the shells appear visually as white while other parts are pale brown) they were re-distributed, 40 left at 32°C and 40 moved to 25°C. At each temperature, half of the specimens were supplemented with 1 ml of exponentially growing monocultures of the three isolated symbionts from *P. calcariformata,* while the other half were considered as the control with no symbiont addition. The water was replaced and supplemented with 1 ml of each symbiont monoculture weekly. Specimens were observed for changes in colour, indicating recovery from partial bleaching that appeared in most treatments after 75 days. At this point, 10 specimens (randomly chosen from two different culturing flasks) from each treatment were immediately frozen at −80°C and stored until further genetic analysis of their algal symbiont community.

### (d) DNA extraction, amplification and sequencing

DNA extractions were performed on samples from the symbiont switching and shuffling experiments following the [35] CTAB method with the following changes. Half the suggested volume of CTAB buffer (Promega, catalogue no. MC1411) and RNase A (QIAGEN, catalogue # 19101) was added directly to a 1.5 ml Eppendorf tube containing the specimen. A sterile pestle was then used to crush the specimen and incubated at 55°C overnight (12–18 h) in a rotating oven. Finally, the DNA was resuspended using Low TE Buffer (Tris-HCl 10 mM pH 8.0, EDTA 0.1 mM pH 8.0) and incubated at 50°C for 30 min in a shaking oven.

The primers TAReuk454FWD1 (5′-CCAGCASCYGCGGTAATTCC-3′) and TAReukREV3 (5′-ACTTTCGTTCTTGATYRA-3′) [36] were used to target the V4 region of the 18S rRNA marker gene. Adaptor sequences for the second-round PCR were added to the primers. Fragments were amplified using QIAGEN Multiplex PCR Master Mix (catalogue no. 206145) in a 20 μl reaction volume. Thermocycling conditions were set at 95°C for 10 min, 20 cycles of 95°C for 30 s, 52°C for 45 s, 72°C for 1 min, and a final step of 72°C for 10 min. PCR products were visualized on a 1% agarose gel. Both extraction and PCR negatives (of both PCR rounds) had no visible amplification on the gel.

First-round PCR products were purified by 1.0× SPRI bead clean-up (AMPure XP bead-based reagent, Beckman Coulter). Second-round PCR was performed with IDT TruSeq Unique Dual Index primers (Integrated DNA Technologies) and KAPA HiFi HotStart ReadyMix (Roche). Thermocycling conditions were set at 98°C for 2 min, 10 cycles of 95°C for 20 s, 65°C for 15 s, 70°C for 30 s and a final step of 72°C for 5 min. Second-round PCR products were purified by 1.0× SPRI bead clean-up (AMPure XP bead-based reagent, Beckman Coulter). Final libraries were quantified by Qubit dsDNA HS Assay (Invitrogen, Thermo Fisher Scientific) and size distribution assessed on Fragment Analyser using HS NGS Fragment Kit (Agilent). Libraries were pooled equimolarly based on measured concentration and average size.

The quantity and quality of each pool was assessed by Bioanalyser and subsequently by qPCR using the Illumina Library Quantification Kit from Kapa (KK4854) on a Roche Light Cycler LC480II according to manufacturer’s instructions. Briefly, a 10 µl PCR reaction (performed in triplicate for each pooled library) was prepared on ice with 6 µl SYBR Green I Master Mix and 2 µl diluted pooled DNA (1 : 1000 to 1 : 100 000 depending on the initial concentration determined by the Qubit dsDNA HS Assay Kit). PCR thermal cycling conditions consisted of initial denaturation at 95°C for 5 min, 35 cycles of 95°C for 30 s (denaturation) and 60°C for 45 s (annealing and extension), melt curve analysis to 95°C (continuous) and cooling at 37°C (LightCycler LC48011, Roche Diagnostics Ltd, Burgess Hill, UK). Following calculation of the molarity using qPCR data, template DNA was diluted to 10 pM and denatured for 5 min at room temperature using freshly diluted 0.1 N sodium hydroxide (NaOH). The reaction was terminated with the addition of HT1 buffer and the libraries sequenced on the Illumina MiSeq platform (Illumina, San Diego, USA) using version 2 chemistry, generating 2 × 250 bp paired-end reads.

## 3. Bioinformatic analysis

After the sequencing process the base-calling and demultiplexing steps were performed by CASAVA 1.8.2, to produce sample sequence files. These files were then imported into QIIME2 (version 2020.8) for the following steps: (i) the PCR primers were removed by using cutadapt and only the sequences showing the presence of the primers were retained; (ii) the obtained raw sequences were denoised by using dada2; (iii) the resulting amplicon sequence variants were assigned to the most likely taxonomy by using sk-learn tool (reference database used Silva 138). The sequence files for the ‘Symbiont shuffling experiment’ and for the ‘Symbiont switching experiment’ were co-analysed within QIIME2, and then the sequence count tables for each experiment were exported. Only diatom sequence larger than 5% summed across all samples were considered and differences between groups were tested using the Permutational Multivariate Analysis of Variance (PERMANOVA) test and subsequent pairwise comparisons.

## 4. Results

### (a) Symbiont shuffling experiment

After 30 days of exposure to the different temperature treatments, almost all specimens were alive as indicated by their pseudopodial activity. All specimens of *P. calcariformata* from all treatments, as well as those of *A. lobifera* from 25°C and 32°C, appeared with their typical brown colour, indicating the presence of their symbionts. *A. lobifera* specimens exposed to 35°C exhibited partial or complete bleaching (as described by the bleaching index of [37]), and at 37°C, the bleaching effect was more pronounced, and five specimens of *A. lobifera* did not survive.

**Figure 2.**
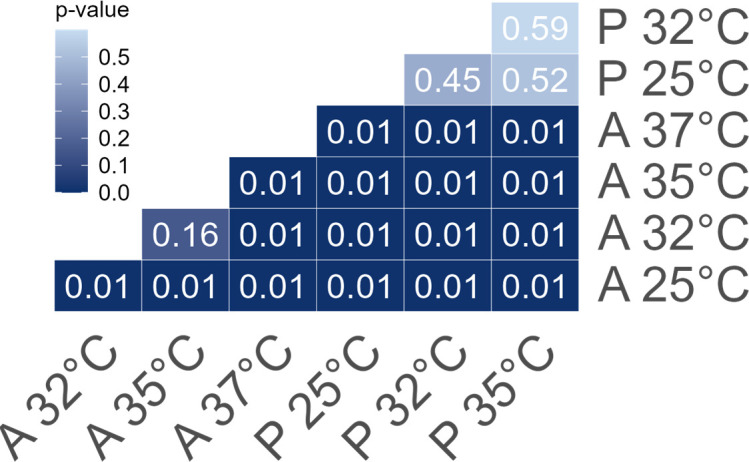
Heat map illustrating the *p*-values of pairwise comparisons between the diatom symbiont communities of both host species across the temperature treatments. A = *A. lobifera* and P = *P. calcariformata*.

**Figure 3.**
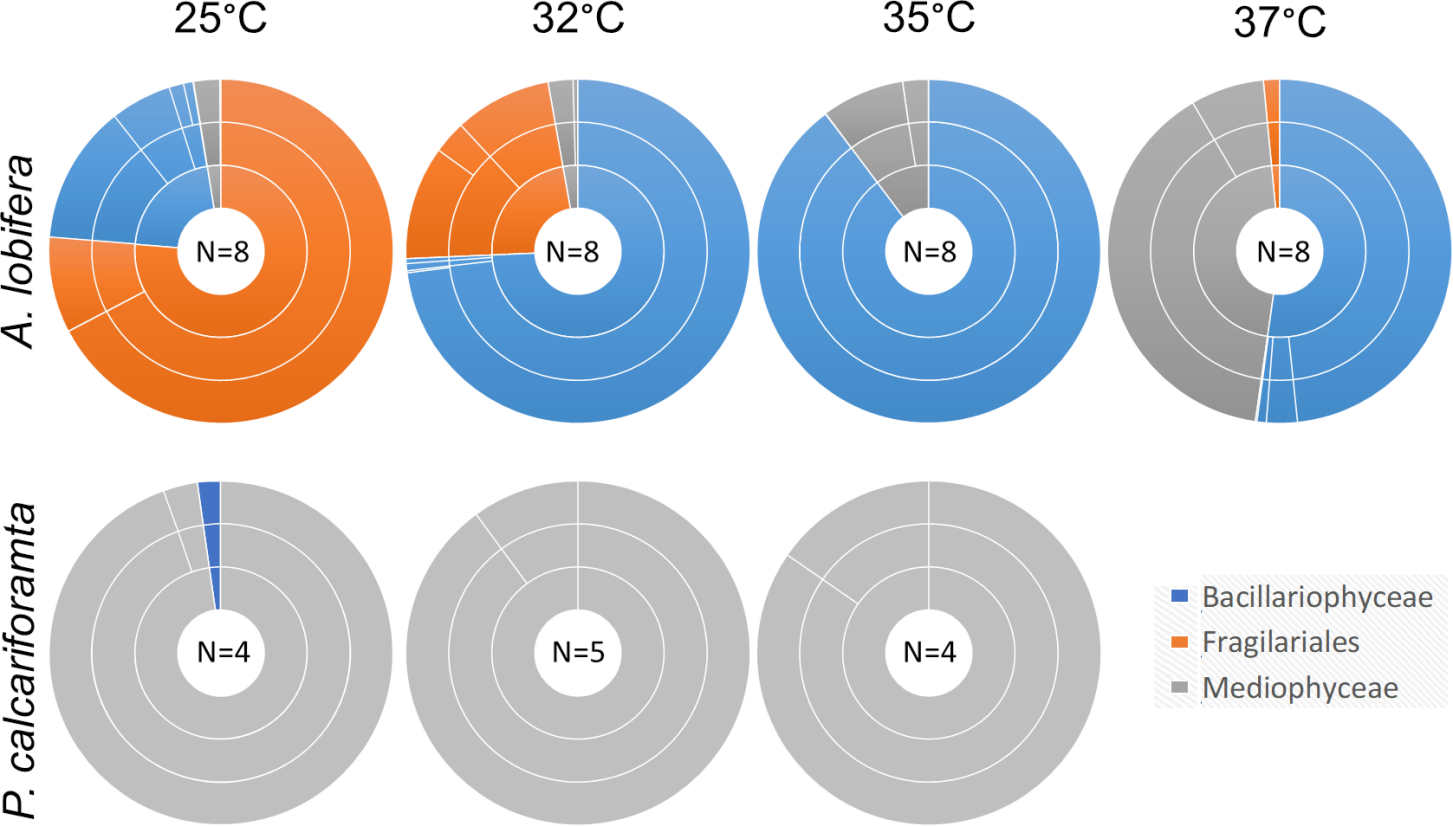
Symbiont community composition of *A. lobifera* (top) and *P. calcariformata* (bottom) after exposure to different temperatures. Inner to outer circles represent the family, genus and species level, respectively.

There was a significant difference in the internal diatom symbiont community between the more heat tolerant *P. calcariformata* and the less heat tolerant *A. lobifera* across different temperature treatments. The significant interaction between host species and temperature indicates that the response was not uniform across the two species (table 1). The diatom symbiont communities in specimens of *P. calcariformata* were not significantly different between the temperature treatments (figure 2), and were all composed of species from the family Mediophyceae, highly dominated by the species *Arcocellulus cornucervis* (56%–99%) (figure 3). One specimen from the 25°C treatment also hosted 8% of the genus *Amphora* from the family Bacillariophyceae which was not present in any of the other *P. calcariformata* specimens.

**Table 1.** PERMANOVA results for the shuffling experiment.

	Df	sum of sqs	*R* ^2^	*F*	Pr(>*F*)
species	1	4.9346	0.4025	49.7922	0.001
temperature	1	2.5649	0.20921	25.881	0.001
species : temperature	1	0.8954	0.07303	9.0347	0.001
residual	39	3.8651	0.31526		
total	42	12.26	1		

Communities of diatom symbionts in *A. lobifera* were considerably more affected by temperature with significant differences between all temperatures (figure 2). Almost all specimens exposed to 25°C were dominated by the family Fragilariaceae and mostly by the species *Serratifera brevis* (63%–98%), and only one specimen was dominated by the family Bacillariophyceae. Specimens exposed to 32°C, were mostly dominated by the genus *Nitzschia* from the family Bacillariophyceae (53–97%), with one specimen dominated by the family Fragilariaceae. Exposure to 35°C also resulted in dominance of the genus *Nitzschia* with much higher relative abundance of 97–100%, with only one specimen that was dominated by a different species from a different family, *Arcocellulus cornucervis*. Specimens exposed to 37°C still had high abundances of the genus *Nitzschia* (18–85%) but also exhibited high abundances of *Arcocellulus cornucervis* (up to 61%; figure 3; electronic supplementary material, table S1).

### (b) Isolated symbionts experiment

**Figure 4.**
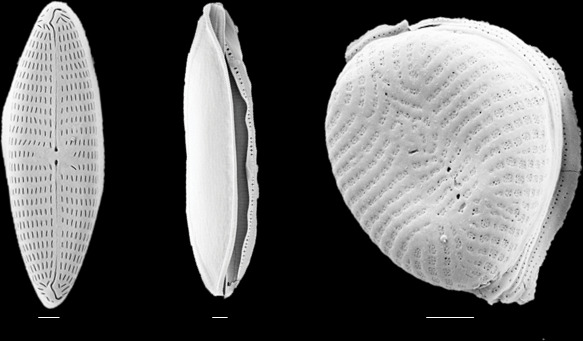
SEM images of isolated symbionts from *P. calcariformata*. From left to right: *Navicula* sp, *Nitzschia* sp. and *Amphora roettgeri*. Scale bars are 1 um.

Isolated symbionts from *P. calcariformata* were identified by morphology as *Amphora roettgeri* and *Nitzschia sp*. from the family Bacillariophyceae and *Navicula sp*., from the family Naviculaceae (figure 4). Both *Navicula sp*. and *Nitzschia sp*. only survived at 25°C with an average growth rate of 1.2 ± 0.5 and 0.8 ± 0.07, respectively. *Amphora roettgeri* survived at all temperature treatments with similar growth rates of 0.95 ± 0.1 at 25°C and 32°C, and 0.9 ± 0.2 at 35°C, so appeared to have the highest thermal tolerance of the isolated symbionts. Unfortunately, *A. cornucervis* was not successfully isolated and its thermal tolerance could not be examined.

### (c) Symbiont switching experiment

**Figure 5.**
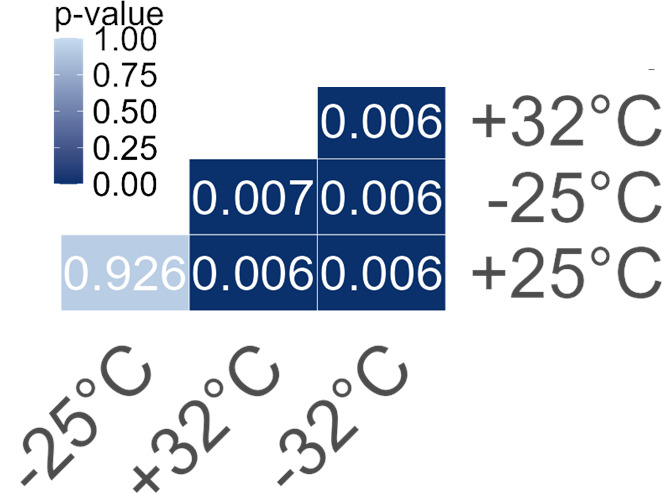
Heat map illustrating the *p*-values of pairwise comparisons between diatom symbiont communities of *A. lobifera* with (+) and without (−) the addition of *P. calcariformata* symbionts to the culturing media at 25°C and 32°C. PERMANOVA between all groups resulted in *F* = 5.231 and *p*‐value = 0.001.

After partial bleaching at 32°C before the switching experiment, *A. lobifera* was reintroduced to 25°C with and without the introduction of *P. calcariformata* symbionts exhibited their typical brownish colour indicating recovery of the symbiont algae in terms of biomass. The diatom symbiont communities in both cases were not significantly different between specimens with and without symbionts added (figure 5) and were composed almost exclusively from the family Fragilariaceae: 71–100% *Serratifera brevis* and 76–96% an unassigned species from the genus Fragilariales. The only exceptions were one specimen from each treatment (with and without the symbiont introduction) that was composed of 100% one species from the family Bacillariophyceae. The similarity to *A. lobifera*’s diatom communities with and without the introduction of symbionts under 25°C, as well as to the 25°C treatment in the shuffling experiment demonstrate recovery from the bleaching to a similar community composition as was originally found within the holobiont i.e. indicative of regrowth after bleaching of the community within the foraminifera.

**Figure 6.**
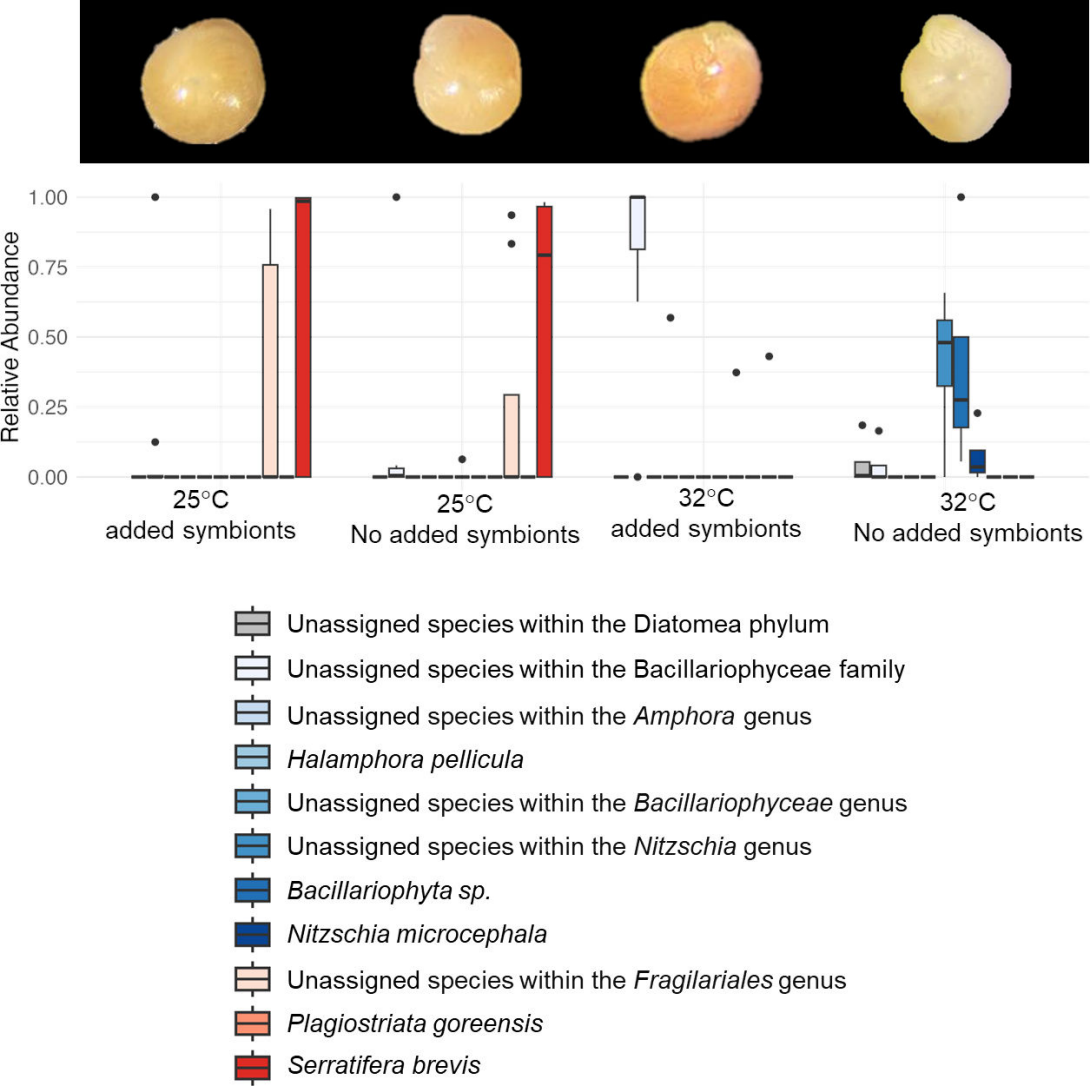
Specimens of *A. lobifera* after exposure to 25°C and 32°C with and without the introduction of symbionts from the families Bacillariophyceae and Naviculaceae isolated from *P. calcariformata*, and the relative abundance of diatom symbionts species within hosts in each treatment. Each bar represents a species according to the colours in the legend but more generally blue colours represent the Bacillariophyceae family and red colours represent the Nitzschia family. Unassigned taxa are not matched to any taxa represented in the database.

After exposure to 32°C, the diatom symbiont communities within *A. lobifera* with and without the introduction of symbionts were significantly different from each other, as well as from the 25°C treatments (figure 5). *A. lobifera* from the control (no added symbionts) were bleached although most specimens were still alive (evident by their active pseudopodia) and dominated by the genus *Nitzschia* (*Bacillariophyta sp*. and an unassigned species totalling 71–100% of the community). By contrast, the partially bleached specimens from the treatment that contained isolated *P. calcariformata* symbionts had gained a dark brownish colour indicating they have taken up symbionts from the culturing media (demonstrated in figure 6). These newly recovered algal communities were mostly (6 out of 7 specimens) 100% of an unassigned species from the family Bacillariophyceae (similar to the isolated introduced symbionts), and one specimen comprised *Halamphora pellicular* (57%) and *Plagiostriata goreensis* (43%). A taxon is considered unassigned if it is absent from the database, indicating that the unassigned taxa are dissimilar from those that are assigned. This suggests that the unassigned species from the family Bacillariophyceae is different than *Bacillariophyta_sp*. and from the unassigned *Nitzschia* species present in the 32°C control (without introduction of symbionts).

## 5. Discussion

Algal symbionts provide much of the energetic demand via carbon and/or nitrogen for their calcifying hosts which utilize the products of photosynthesis. Carbon flux is primarily governed by the rates of photosynthesis and respiration, but generally photosynthesis as a biochemical process appears to have a lower thermal optimum than that of respiration [38,39]. In photo-symbioses, the consequence of a decline in the ratio between photosynthesis to respiration of the symbiont may cause a reduction in photosynthate availability that will probably cause a detrimental effect for the host, and more specifically on its ability to meet the energetic demand for calcification. Thus, the photosynthesis : respiration ratio which is proxied by the thermal tolerance of the algal symbionts can either ameliorate or exacerbate the challenges posed by altered environmental conditions. Changes in the community of algal symbionts towards higher abundances of more tolerant symbionts can enhance thermal tolerance of the host [5]. However, endosymbiotic flexibility does not always lead to better resistance to thermal stress and low symbiont flexibility does not necessarily create vulnerable hosts [40]. Most of the knowledge regarding symbiont shuffling and switching stems from coral research and thus is limited to Symbiodiniaceae. Our investigation of diatom bearing LBF suggests that differences in the symbiont community and the ability to adjust it, may contribute to the different thermal sensitivities of the host species.

Changes in the internal diatom community composition with increasing temperature of *A. lobifera* demonstrate shuffling on the family level, shifting from dominance of Fragilariaceae at 25°C, to Bacillariophyceae at 32°C and 35°C, and to co-dominance of Bacillariophyceae and Mediophyceae at 37°C. This transition shows that the diatom symbiont community only resembles that of the highly thermally tolerant *P. calcariformata*, which primarily consists of Mediophyceae, under acute conditions. The symbiont community of *P. calcariformata* hardly changed between the temperature treatments which helps explain the previous observation of maintaining photosynthetic performance up to at least 35°C [29]. A study that characterised the internal symbiont community of *P. calcariformata* from four locations including in an anthropogenic thermal disturbance, identified 17 diatom species. The diverse internal pool of symbionts was suggested to be the key to the thermal resilience of the host, as more functionally relevant members of the diatom community could become more dominant with environmental change [21]. In our study, specimens of *P. calcariformata* in all temperature treatments were overwhelmingly dominated by *A. cornucervis*, which suggests that the tolerance of *P. calcariformata* was not conferred by diversity of the symbiont pool as previously suggested. This also explains the observation that in all four locations studied in [21] *Minutocellus polymorphus* (another species from the Mediophyceae family) was the most common symbiont across all locations. The difference between the diatom community composition between this study and Schmidt *et al*. [21] could be the result of collection in different seasons or somewhat different conditions before the actual experiment (e.g. while transported or while culturing previously to the experiment), however, the observation remains similar that in both studies *P. calcariformata* is dominated by one species of Mediophyceae.

Interestingly, *A. lobifera* substantially changed the symbiont community between temperature treatments but was still affected by bleaching albeit with the presence of *A. cornucervis*. This indicates that the ability to shuffle the community might not be sufficient to extend the thermal tolerance of all hosts, even with the presence of the ‘right’ species perhaps due to differential host demand for carbon and/or nutrients. Moreover, this shuffle only occurred at 37°C, which is higher than thresholds for photosynthesis and calcification (32°C [17,29,41]). While it remains unclear whether foraminiferal host metabolism or internal regulation influences which symbionts are retained, the differing shuffling patterns observed between the two foraminiferal hosts, despite both harbouring a common symbiont (i.e. *A. cornucervis*), suggest that symbiont selection may be actively regulated by the host rather than being solely dictated by the fitness of individual symbiont strains under specific conditions.

An alternative mechanism through which changes in the symbiont composition could confer thermal tolerance is the uptake of new symbiont partners from the ambient water while positively selecting for the more adapted species to changes in environmental conditions (i.e. the adaptive bleaching hypothesis [42,43]). This phenomenon referred to as ‘switching’ of symbionts is well documented in coral larvae and juvenile individuals with recent reports also in adult corals [9,10,44] but was not observed in foraminifera.

Our results clearly show that when under thermal stress the presence of diatoms isolated from a more thermally tolerant host (i.e. *P. calcariformata*) substantially increased *A. lobifera*’s ability to recover from bleaching, evident by their dark brownish colour compared with bleached specimens maintained at the same temperature (32°C) but without the introduction of new symbionts. Further, unlike in *A. lobifera* exposed to 25°C, the diatom community composition of specimens exposed to 32°C (both with and without the introduction of new symbionts) was dominated by the family Bacillariophyceae, but the species composition significantly differed when symbionts were introduced to the culturing media. This suggests that even though shuffling in higher temperatures occurs at the family level, the recovered symbiont community is in fact of a specific species, suggesting that the symbiont’s ability to recover stems from changes at the species level. This is also suggested by the single-species dominance in *P. calcariformata* (*M. polymorphus* in [21], and *A. cornucervis* in this study) which exhibits high thermal resilience. Future studies should attempt to isolate and culture *A. cornucervis* and *M. polymorphus* to include in such switching experiments to examine their specific ability to confer the holobiont with higher thermal tolerance.

This study provides the first direct evidence of exogenous symbiont enhancing the thermal tolerance of the holobiont foraminifera which could demonstrate the foundations of an approach to help mitigate the impact of future warming on LBF. In a natural setting where a high diversity of potential symbiont partners are present, such ability might be highly valuable especially if considering the short generation time of algal symbionts, in comparison with their calcifying host that can allow them to evolve more rapidly, adapt to increasing temperatures and support the energetic needs of the host as it requires more time to adapt.

Our results also show that when the environmental stressor is removed, in our case the return to optimal temperature, the symbiont community can recover and return to a similar community composition as before the bleaching event. The similar response with and without the introduction of exogenous diatoms implies that the ability to recover from a temporary heat stressor such as those imposed by heatwaves, is dependent on the internal pool of symbionts, while the ability to acclimate to long term heat is in symbiont switching (i.e. preferential take up this new symbiont from the ambient water only occurred when under thermal stress).

Our observations regarding the recovery of algal symbiont communities provide a plausible explanation for the mechanism behind the past success of LBF under abrupt climate change in the geological record, as well as an optimistic view on their fate under future warming. Moreover, the approach of independently experimenting on isolated symbionts and hosts showcase a tool to further investigate how symbiont algae supports calcification of highly thermotolerant calcifiers.

## Data Availability

Sequencing data is deposited in the European Nucleotide Archive and available here: https://www.ebi.ac.uk/ena/browser/text-search?query=Symbiont%20switching%20boosts%20heat%20tolerance. Supplementary material is available online [45].
